# Leveraging type 1 diabetes human genetic and genomic data in the T1D knowledge portal

**DOI:** 10.1371/journal.pbio.3002233

**Published:** 2023-08-10

**Authors:** Parul Kudtarkar, Maria C. Costanzo, Ying Sun, Dongkeun Jang, Ryan Koesterer, Josyf C. Mychaleckyj, Uma Nayak, Suna Onengut-Gumuscu, Stephen S. Rich, Jason A. Flannick, Kyle J. Gaulton, Noël P. Burtt

**Affiliations:** 1 Department of Pediatrics, University of California San Diego, La Jolla, California, United States of America; 2 Metabolism Program, The Broad Institute of Harvard and MIT, Cambridge, Massachusetts, United States of America; 3 Program in Medical and Population Genetics, The Broad Institute of Harvard and MIT, Cambridge, Massachusetts, United States of America; 4 Center for Public Health Genomics, University of Virginia, Charlottesville, Virginia, United States of America; 5 Division of Genetics and Genomics, Boston Children’s Hospital, Boston, Massachusetts, United States of America; 6 Department of Pediatrics, Harvard Medical School, Boston, Massachusetts, United States of America

## Abstract

This Community Page presents the type 1 diabetes (T1D) Knowledge Portal, an open-access resource for hypothesis development and target discovery in T1D that aims to help address the challenge of translating genetic discoveries into mechanistic insight.

## Teaser

To address the challenge of translating genetic discoveries for type 1 diabetes (T1D) into mechanistic insight, we have developed the T1D Knowledge Portal (T1DKP), an open-access resource for hypothesis development and target discovery in T1D.

## Introduction

The etiology of type 1 diabetes (T1D), a complex disease characterized by autoimmune destruction of pancreatic beta cells, is incompletely known [1]. There are currently no cures or effective prevention strategies, and only recently has an immune intervention to delay T1D onset been FDA approved (teplizumab) [2]. In the absence of full blockage of T1D initiation and progression to clinical disease, the only treatment is life-long insulin therapy. There is therefore a pressing need to identify new targets for therapeutic intervention. Discoveries from genetic association studies of complex diseases such as T1D can offer novel insight into pathogenesis, reveal potential therapeutic targets [3], and provide human genetic support for preexisting targets [4].

There are major barriers, however, to translating genetic discoveries into biological and therapeutic insights. The results of genetic association studies are inaccessible to many scientists, since utilizing and interpreting large genetic “summary” files requires expertise in data manipulation and knowledge of domain-specific bioinformatics tools. In addition, most T1D risk variants map to noncoding sequence, where detailed functional annotation of the genome is necessary to predict affected cell types and genes [5]. Finally, testing variant and gene function in cellular and animal models remains a substantial undertaking, often requiring years of work.

Here, we report the T1D Knowledge Portal (T1DKP), an open-access resource developed to help advance T1D research by democratizing access to genetic, genomic, and epigenomic data. The primary goal of the T1DKP is to facilitate the generation of accurate, testable hypotheses from T1D genetic association data by providing a user-friendly interface where researchers can view the results of analyses integrating genetic and functional annotation data using contemporary bioinformatic tools, access “curated” resources such as candidate gene lists generated by domain experts in T1D, and query and visualize data for specific variants, genes, regions, and phenotypes. The T1DKP resides within a larger Knowledge Portal Network of disease-specific portals, all based upon the Human Genetics Amplifier (HuGeAMP) software infrastructure.

## Features of the T1DKP

**Fig 1:**
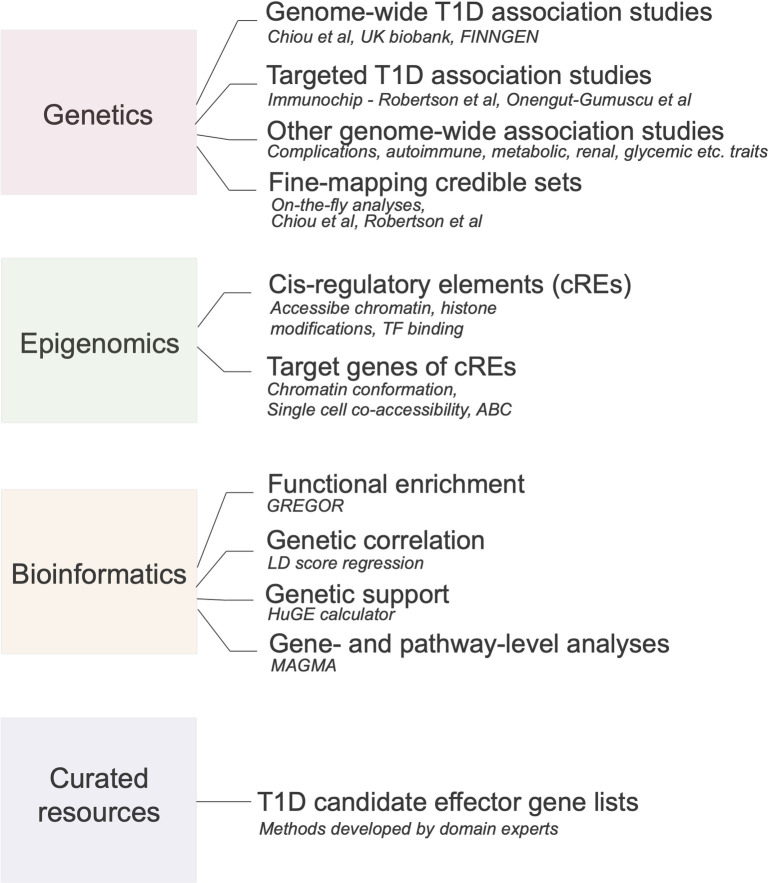
Data content of the T1DKP. The T1DKP provides genetic and genomic data, pre-computed bioinformatics results, and expert-curated resources such as candidate gene lists to the T1D community.

The T1DKP (RRID:SCR_020936), as of June 2023, includes 11 genetic association studies for T1D, including genome-wide association studies (GWAS) from large meta-analyses [6], GWAS from biobanks such as FinnGen, and targeted, fine-mapping studies using the ImmunoChip [7] (Fig 1). The T1DKP also includes 189 association datasets representing 161 T1D-relevant traits, such as diabetic complications, other autoimmune diseases, and glycemic, lipid, renal, and anthropometric traits. We aim to collect all association studies of T1D and relevant phenotypes with available summary statistics by systematically searching the GWAS Catalog, biobanks, and biomedical literature, as well as engaging with the T1D community. We also accept results from manuscripts under review or pre-prints, although these are labeled as “pre-publication” in the T1DKP.

The T1DKP aggregates 5,580 functional annotation datasets from the Common Metabolic Diseases Genome Atlas that describe the location of candidate *cis*-regulatory elements (cCREs) in the human genome and predicted target genes of cCREs in 200 tissues, primary cells, cell lines, and stem cell-derived models. These annotations are collected both from resources such as ENCODE and from studies performed by individual investigators. In the latter case, studies of T1D-relevant cell types are prioritized for inclusion; for example, there are data identifying cCREs in immune cells in baseline and stimulated conditions [8], as well as chromatin interactions linking cCREs to putative target genes in immune cells [9]. Future releases will incorporate additional annotation types currently lacking from the resource, such as molecular quantitative trait loci (QTLs).

The T1DKP web interface includes pages that summarize genetic associations and functional annotations for specific variants, genomic regions, genes, and phenotypes. Visualizations on these pages, such as PheWAS forest plots and LocusZoom association plots [10], facilitate user interaction with genetic data. Results from bioinformatic methods integrating genetic and genomic datasets provide additional insight. For example, the gene page includes genetic support analyses that indicate whether the gene is likely involved in a trait [4,11]. In another example, the phenotype page includes analyses that describe functional annotations in different cell types and tissues enriched for trait-associated variants [12] and biological pathways associated with the trait [11]. Several interactive modules can also be accessed from summary pages to enable more detailed investigation. Finally, the T1DKP facilitates independent investigations by providing all genetic and functional annotation datasets for download or programmatic access via a REST API (available at http://bioindex.hugeamp.org). Each page and tool of the T1DKP is documented with available online tutorials and videos.

**Fig 2:**
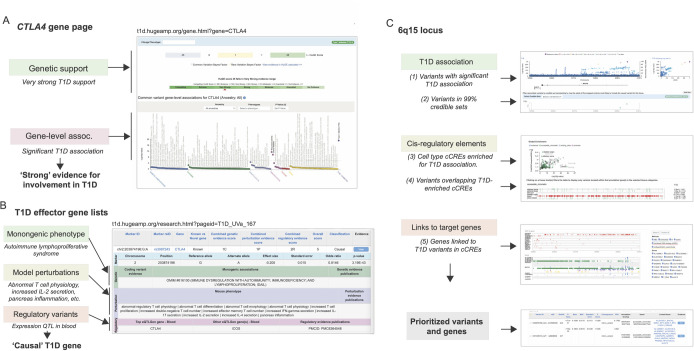
Distilled evidence supporting T1D variants and candidate genes in the T1DKP. The T1DKP provides distillations of human genetic results for researchers. (A) The summary page for the *CTLA4* gene provides evidence that this gene affects T1D risk, including results providing “very strong” support from the HuGE calculator and strong evidence for T1D association from MAGMA. (B) A “T1D effector genes” list predicts *CTLA4* as a “causal” gene for T1D based on genetic, perturbation, and gene regulatory evidence. (C) Predicting causal mechanisms at the 6q15 locus. (top) Prioritizing variants with evidence for affecting T1D risk based on significant association and 99% credible sets. (middle) Prioritizing variants overlapping cCREs active in T1D-enriched cell types and tissues. (bottom) Prioritizing genes linked to variants in cCREs in specific cell types and tissues. From these analyses, 2 variants are predicted as causal candidates for T1D at this locus, which are linked to multiple candidate genes including *BACH2* in immune cells.

For researchers who are not experts in human genetics, the T1DKP offers intuitive summaries of genetic results. On the gene page, the level of genetic support for a gene across all datasets in the T1DKP is shown qualitatively, ranging from “Compelling” to “No evidence” (Fig 2A). On a separate page, expert-curated candidate gene lists are provided, accompanied by supporting evidence such as protein-coding mutations causing T1D-relevant monogenic phenotypes, noncoding T1D variants linked to the gene, and model system perturbations causing T1D-relevant phenotypes (Fig 2B). These lists and supporting evidence are designed to be used by non-geneticists to develop hypotheses and guide experiments for specific genes. For researchers wishing to explore the details of genetic and genomic data in greater detail, the T1DKP provides interfaces and tools that can help to prioritize candidate genes likely involved in T1D risk at specific loci. For example, from the region page the user can link to a “Variant Sifter” module that enables selection of a series of filters to prioritize candidate variants, genes, and tissues/cell types to guide experiments in that region (Fig 2C).

## Conclusion

The T1DKP enables exploration of genetic and functional annotation data relevant to T1D on an interactive website designed for use by both experimental biologists and experts in human genetics. Compared to disease-agnostic resources that also provide platforms for analyzing human genetic and genomic data such as Open Targets, 2 core strengths of a disease-focused resource such as T1DKP are aggregation of datasets from studies of high value to that specific disease that may be missing from “pan-disease” catalogs and incorporation of curated datasets created by domain experts. Consequently, the T1DKP primarily focuses on traits directly related to T1D, and users who wish to view associations for a wider range of traits should consult other portals in the Knowledge Portal Network, including the Association to Function Knowledge Portal, or resources such as the GWAS Catalog and Open Targets.

Moving forward, a key goal of the T1DKP is to continue engaging with the T1D community to identify and add T1D-relevant datasets, as well as to generate new datasets from available cohorts. For example, association data from whole genome and exome sequencing will help identify genes carrying rare variants involved in T1D; association data from different ancestries will both reveal additional T1D risk and help resolve causal variants for signals shared across populations; functional annotations such as molecular QTLs and systematic screens of variant function will enhance interpretation of risk loci; and gene perturbation phenotypes in human cells and model organisms will facilitate understanding gene function in T1D. We also will continue to improve expert-curated candidate gene lists, which is a unique aspect of this resource to our knowledge, by collaborating with a wider range of researchers and incorporating additional data types. We look forward to collaborating with the T1D community to advance these and other areas of the T1DKP.

## Abbreviations

cCRE: candidate *cis*-regulatory element
GWAS: genome-wide association studies
HuGeAMP: Human Genetics Amplifier
QTL: quantitative trait locus
T1D: type 1 diabetes
T1DKP: T1D Knowledge Portal
